# Conceptualising the social in mental health and work capability: implications of medicalised framing in the UK welfare system

**DOI:** 10.1007/s00127-023-02449-5

**Published:** 2023-03-13

**Authors:** Annie Irvine, Tianne Haggar

**Affiliations:** 1 ESRC Centre for Society and Mental Health, King’s College London, London, UK; 2 The Policy Institute, King’s College London, London, UK

**Keywords:** Mental health, Distress, Entification, Medicalisation, Welfare, Unemployment

## Abstract

**Purpose:**

This paper asks whether the separation of mental health from its wider social context during the UK benefits assessment processes is a contributing factor to widely recognised systemic difficulties, including intrinsically damaging effects and relatively ineffective welfare-to-work outcomes.

**Methods:**

Drawing on multiple sources of evidence, we ask whether placing mental health—specifically a biomedical conceptualisation of mental *illness* or *condition* as a discrete agent—at the core of the benefits eligibility assessment process presents obstacles to (i) accurately understanding a claimant’s lived experience of distress (ii) meaningfully establishing the specific ways it affects their capacity for work, and (iii) identifying the multifaceted range of barriers (and related support needs) that a person may have in relation to moving into employment.

**Results:**

We suggest that a more holistic assessment of work capacity, a different kind of conversation that considers not only the (fluctuating) effects of psychological distress but also the range of personal, social and economic circumstances that affect a person’s capacity to gain and sustain employment, would offer a less distressing and ultimately more productive approach to understanding work capability.

**Conclusion:**

Such a shift would reduce the need to focus on a state of medicalised incapacity and open up space in encounters for more a more empowering focus on capacity, capabilities, aspirations, and what types of work are (or might be) possible, given the right kinds of contextualised and personalised support.

## Introduction

The UK operates a conditionality-based welfare benefits system for people of working age. For those seeking income-replacement benefits on the grounds of ill health, the level of expectation to take active steps towards work depends on an assessment of health-related functional impairment, termed the Work Capability Assessment (WCA).^1^ Introduced in October 2008, the WCA was a key component of wider reforms to health-related benefits. It accompanied the introduction of Employment Support Allowance (which replaced the predecessor Incapacity Benefit and has since been superseded by Universal Credit) and signalled an increase in the levels of conditionality and requirement for work-focussed activity applied to people with health-related claims. Although based on the premise that work can have wellbeing benefits and that health conditions should not necessarily be seen as a barrier to work, the WCA “has however been controversial from the outset” [1]. A large body of qualitative research consistently finds that, for people with health problems, the WCA is experienced as distressing, intrinsically harmful, and ineffective as a mechanism to accurately determine their capacity for work [2, 3, 4, 5, 6, 7, 8, 9, 10, 11, 12, 13]. As we elaborate below, these problems are particularly profound for people experiencing mental health difficulties.

In this paper, we argue that it is the centrality of *health* and the dominance of a de facto *medicalised framing* of distress within the current benefits assessment system that renders it particularly problematic in the case of mental health. This framing invites only one way of explaining why working is not possible—that is, the functional impairment effects of a “health” condition. The problem has three dimensions. First, positioning a mental health problem as a discrete entity makes it difficult for claimants to convey the particular nature of “functional impairment” imposed by their distress. Second, the process decontextualises assessment of function from any specific work/employment environment. Third, the medicalised lens leaves unacknowledged the complex web of social circumstances that render a person unable to engage in paid employment at a given time.

In this paper, we ask whether this separation^2^ of mental health from its wider social context during the WCA processes is a contributing factor to well-recognised systemic difficulties of distress, harm and ineffective intervention. We ask whether placing mental health—specifically a biomedical conceptualisation of mental *illness* or *condition* as a discrete agent—at the core of the benefits eligibility assessment process presents obstacles to (i) accurately understanding a claimant’s lived experience of distress (ii) meaningfully establishing the specific ways it affects their capacity for work, and (iii) identifying the multifaceted range of barriers (and related support needs) that a person may have in relation to moving into employment. We explore whether a more socially informed approach to the WCA might facilitate less distressing, more meaningful, and ultimately more productive experiences for claimants with mental health problems.

The focus of this article is specifically the WCA. We acknowledge that in later parts of the claimant journey within the UK system, there have been efforts to introduce more holistic and personalised approaches to determining capacity for work-related activity. For example, conversations between Work Coaches and claimants (in ongoing appointments after the award of benefits) are intended to facilitate negotiation of “personalised conditionality” [14, 15] whereby the extent and specific type of work-related activities a claimant commits to are tailored to their wider personal circumstances and constraints. Notwithstanding the extent to which this personalisation is being achieved in practice [13, 16, 17], we would assert that—as the essential “gateway” to these subsequent stages of the benefits process—the absence of such holistic and socially informed approaches *during the WCA itself* remains a critical obstacle to effective support and trust in the system.

This paper is an exploration and a provocation. Its data sources are multiple and selective, aiming to construct a new proposition for discussion rather than demonstrate empirical findings. Our argument has developed through a combination of our own past and ongoing qualitative research with benefit claimants and mental health employment support organisations, as well as extant research in the fields of social policy and mental health, which reports the experiences of claimants and frontline welfare advisers.

## Health assessment in the UK welfare system

People seeking to have their health circumstances taken into account when applying for working-age benefits must complete a specific form, the UC50. In most cases, claimants are then required to undergo a Work Capability Assessment (WCA), conducted by an independent assessor, to establish how and to what extent their health condition(s) limit capacity for work. The WCA outcome determines which of three benefit sub-groups the claimant will be assigned to. In the current system these are: *fit for work*, *limited capability for work*, and *limited capability for work and work-related activity*. As the names suggest, these groupings have different degrees of requirement for active jobsearch and work preparation. People in the *fit for work* and *limited capability for work* groups are required to undertake jobsearch and/or other “work-related activities”, as agreed with their Work Coach, and face the risk of sanctions (financial deductions) from their benefits if they fail to do so. Claimants in the *limited capability for work and work-related activity* group are not expected to seek work nor to prepare for work in the future.^3^

**Fig. 1:**
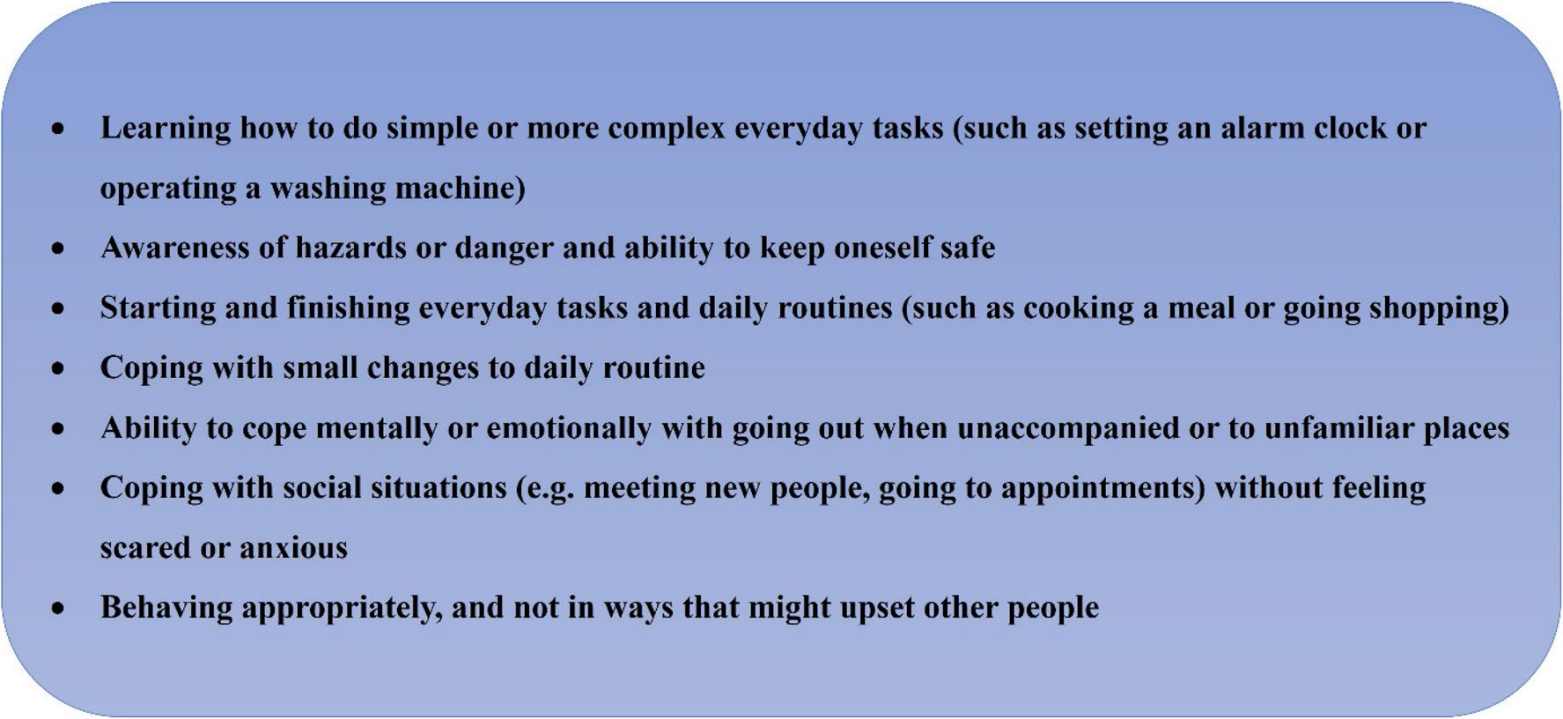
Descriptors for mental, cognitive and intellectual capability (UC50)

Significantly, the WCA is expressly not based upon establishing the *presence or absence* of a given health condition. The focus is on determining degree of *functional impairment*, i.e. the *effect* that any health condition has on a person’s capacity to undertake paid work. A series of 18 “descriptors” are used to assess physical, mental, cognitive and intellectual functions, with points allocated according to severity of limitation in each aspect. Figure 1 summarises the domains of function within the UC50 pertaining to the impacts of “mental, cognitive and intellectual capabilities”.^4^

A key problem faced by people experiencing mental health problems is that the descriptors used to determine type and degree of functional impairment are widely perceived as inadequate in their ability to reflect and evaluate the impact of mental ill health on a person’s capacity to find and sustain employment [18]. Difficulties include a weighting towards physical impairments, limited scope to convey the impact of fluctuating conditions and doubts about the expertise of assessors in recognising and understanding mental health conditions [3, 7, 10, 19]. Despite the inclusion of questions about psychological or emotional difficulties (see Fig. 1), qualitative research consistently finds that the current system does not enable claimants to accurately or adequately explain the specific ways in which mental health problems constrain their capacity for work, with claimants reporting experiences of dismissal and misrepresentation of their accounts [3, 5, 6, 7, 8, 9, 10, 13]. This has further detrimental effects, because the experience of feeling disbelieved, judged and misunderstood exacerbates claimants’ distress. Whilst the WCA seeks to determine the *effects of* a mental health condition that constitute work limitations, rather than the veracity of the condition itself, this nuance is generally lost; to assess the *effects* of the condition is taken as to evaluate the legitimacy of the condition itself [13]. Thus, for the claimant who “fails” the WCA, the conclusion that their suffering is not deemed a barrier to work is interpreted as their suffering not believed to be real. As described by an employability keyworker in Irvine et al. [20]:

“What you’re saying is, if I don’t qualify, I don’t have a mental illness. That’s what you’re saying really: that I’m well … And I think that really upsets people, because to actually say to somebody, ‘You’re not poorly’, when they’re dealing on a daily struggle with whatever they’re dealing with, that’s really offensive … If they’re told ‘no’, it's like they've opened themselves to their worst possible personality problems that they really struggle with, and they’re told—slapped in the face almost—told there's nothing wrong with you.”

In addition to constraining claimants’ ability to convey the effects of mental distress, the WCA is also an abstract assessment, which neglects to recognise the context-specific nature of work ability. Moreover, it takes a narrowly health-focussed lens on work limitations, which diverts attention from the contextual and antecedent social factors that underlie mental distress and the multiplicity of interrelated circumstances which may need attention and support in order for someone to think productively about engagement with employment.

In the following sections, we develop our proposition that the decontextualization and separation of a medically framed “mental health condition” as the dominant driver of work incapacity is problematic in three senses: (i) entifying a mental health condition as a discrete agent (ii) decontextualising assessment of work function from any specific work environment and (iii) isolating mental health from the broader range of socioeconomic factors that affect work capacity where someone is (also) experiencing mental distress. Together, we argue, these three separations help to explain the challenges faced by benefit claimants in conveying their lived experience of work-related limitation.

## Separation of mental distress from social context: three challenges

### Entification of “mental illness” as a discrete agent

The first challenge stems from treating mental health problems as discrete entities, separate from their functional effects. Brinkmann [21] (citing Valsiner [22]) writes of the “entification” of mental health problems, i.e. treating a psychological “condition” as an independent explanatory agent: “Entification involves transforming a trait, temperament, emotion or some other psychological phenomenon into a ‘thing’, typically with causal powers to affect action” [21].

The positioning of mental illnesses as distinct and agentic medical entities is apparent in language used in the UC50 application form, which refers to “your *disabilities, illnesses or health conditions*” and “problems you may have *from* mental illnesses like schizophrenia, depression and anxiety” (emphasis added). These forms of words implicitly signify a biomedical conceptualisation of mental health conditions/illnesses as discrete entities, which *in turn* cause functional problems to the individual [23]. However, critical psychiatry has argued that mental health diagnoses are tautologous; in the absence of discrete biomarkers of mental illness [24], diagnostic categories are based only on symptoms, and symptoms become the basis of diagnosis [25, 26, 27]. In a parallel vein, Rose [28] notes that the social contexts of mental distress “are not external to the disorder … They are constitutive of the complaint”. Thus, the difficulties that claimants experience in clearly conveying the effects *of* their “mental health condition” may be because the effects *are* the mental health condition. These circularities inherent in conceptualising psychological distress as a “health” issue may be one factor in the difficulties claimants experience in conveying the nature of functional impairment posed by a mental health “condition”. As noted by Gipps [29] in relation to anxiety, it may be a mistake to “treat anxiety as a problem in its own right, rather than as a useful indicator that an as-yet insufficiently met existential challenge is being encountered.”

Manifestations of mental distress that may reduce people’s ability to function reliably and productively in work include weariness, dizziness, sleep problems, tearfulness, social withdrawal, irritability, volatility, panic attacks, slowed thinking and difficulties with concentration [30, 31, 32, 33, 34, 35]. However, these challenges are not easily captured by the WCA’s questions around coping and behaviours in broadly defined “social situations” or when “going out” because many functional effects of mental distress manifest *within and through* social interactions. This brings us to our second proposition, that work-limiting effects of mental health problems cannot be evaluated outside of the context of a specific work environment.

### Decontextualisation from specific work environments

The WCA is conducted in a way that is both physically and conceptually decontextualised from any specific work context. This separation is fundamentally problematic because being “fit for work” is not an all or nothing distinction and depends on the specific interpersonal and occupational demands of any given job role [36, 37, 38]. Work functioning is “associated with, but is not merely a consequence of, the condition … [There is] a dynamic interaction between personal resources and symptoms, situated job tasks and the social environment at and outside work” [39]. As observed by an employment service manager in Bonfils’ study [40], “work capacity is not just something you have; it is something you can develop and it depends on the setting or place in which you are employed”. These issues signal the importance of “real world” assessment of work capacity [41, 42, 43]. However, in the current system, assessors make a decontextualised assessment of capacity to work. Mental health is not “unpacked” in terms of the specificity of (fluctuating) impacts and the context-dependency of employment support needs.

Furthermore, the critical importance of line manager and co-worker support to sustaining employment alongside ongoing mental health problems has been established through quantitative and qualitative research [44, 45, 46]. These are factors that cannot be predicted prior to job entry. Social relationships within the workplace, and strong “person-environment fit” are fundamental to the recovery and maintenance of work function [38, 46, 47]. Reflecting on the UK’s legal frameworks around supporting workplace mental health, Almond et al. [48] note the flaws inherent in treating mental ill health as “something that exists independently of the workplace, rather than something which may be created or exacerbated or, sometimes, improved by it”.

The fact that many people sustain paid work alongside mental health problems [33, 39, 49, 50] indicates that mental health is often not the determining factor in whether someone is able to work or not; workplace and personal relationships, caring responsibilities, physical health, education, skills, finances, housing, as well as local labour markets and employment security, all influence the sustainability of employment alongside mental health problems [46, 51, 52]. This brings us to our third proposition; that recognition of wider social context is crucial to a holistic understanding of a person’s capacity for work.

### Isolation of mental health from wider contextual barriers to work

Institutional acknowledgement of restricted work capacity currently depends upon the framing of work limitations as determined by *health* conditions. However, people claiming out-of-work benefits frequently experience a multitude of interwoven challenges that may include: insecure housing, lone parenthood, caring responsibilities, domestic violence, offending, substance use, debt, low literacy, lack of qualifications, limited work experience, rural isolation, limited local employment options, the compounding effects of long-term unemployment on confidence and self-esteem, as well as physical and/or mental health problems [53, 54, 55, 56, 57, 58]. All these factors are influential in a person’s cognitive and practical capacity to seek, secure and sustain paid employment [54].^5^

McManus et al. [61] describe claimants for whom “issues were multifaceted, complex and had occurred over the life course” where “poor mental health was not necessarily the primary problem faced, but was a compounding problem”. Similarly, Hudson et al. [62] found that, whilst health conditions were perceived as a primary barrier to work by a “substantial minority” of claimants, a wide range of other “realistic concerns” were raised, including financial insecurity, finding appropriate work, lack of qualifications, age discrimination and local labour market conditions. “Employability” is a multifaceted combination of individual factors, personal circumstances and external factors operating from both the supply and demand sides [63].

Mental health problems are causally correlated with social stress, adversity and trauma [64, 65, 66]. Qualitative research [33, 54, 61, 62, 67, 68] shows that people identify and locate the *source* of their distress in social circumstances, including job loss, relationship breakdown, problem debt, housing problems, physical illness, abuse and bereavement. From a work capability perspective, these findings are important because these social drivers of distress may be equally, if not more significant barriers to work as the mental health “condition” itself. Yet the WCA as currently designed does not provide space for a more holistic range of work-limiting circumstances to be described or taken into account. The centralising of mental health conditions as the driver of worklessness detaches distress from the social circumstances that underlie and produce it, and which may be the more fundamental barriers to work. In the words of one participant in Ploetner et al. [8], “*They need to look at the whole picture… they need to be holistic and they are not.*”

## Discussion

**Fig. 2:**
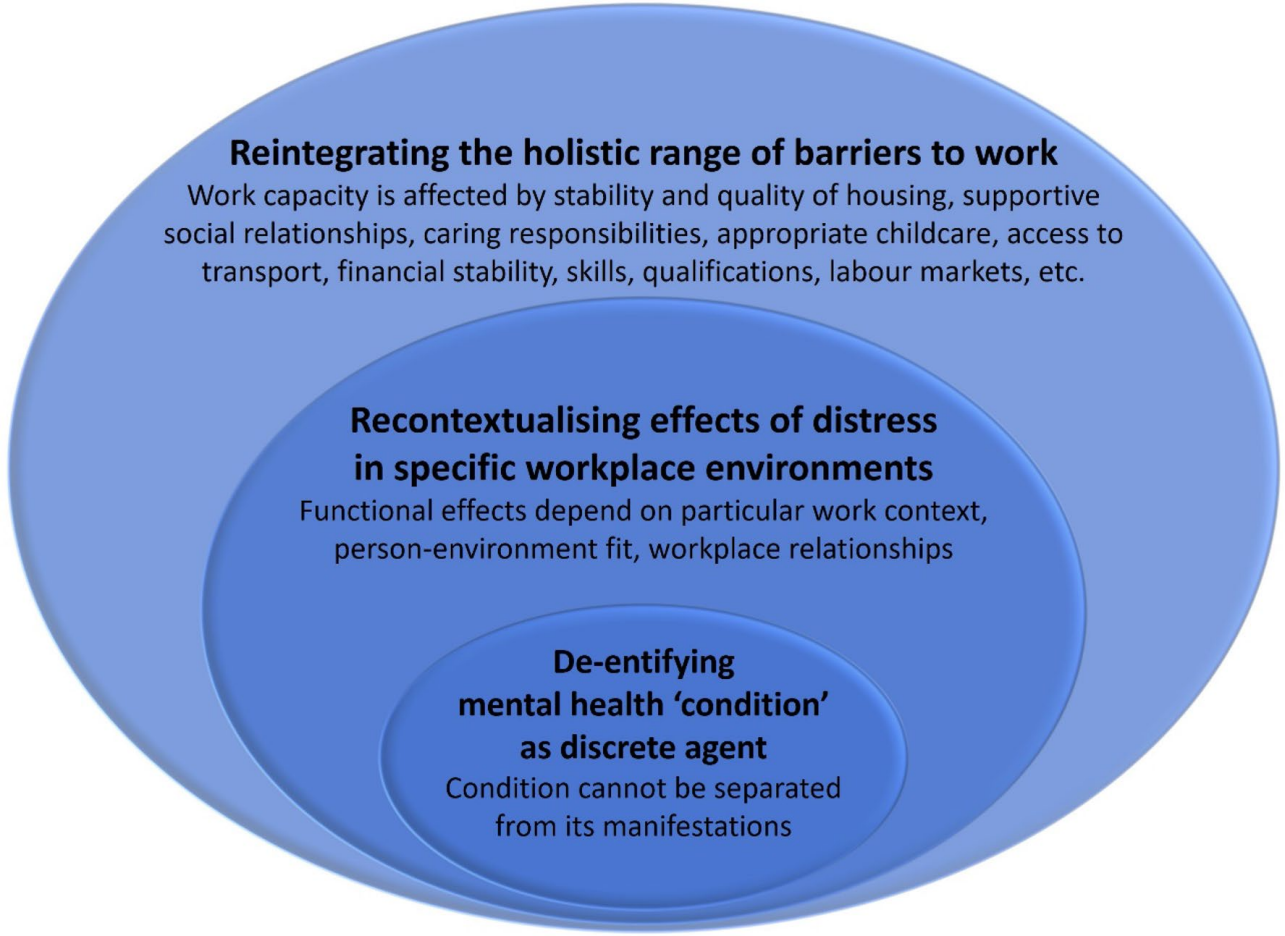
Reintegrating separations within the Work Capability Assessment

In this paper, we have argued—with a particular focus on the assessment of work capability—that mental health problems cannot be viewed as discrete and context-free entities. They arise, manifest and shape people’s lived experiences *in and through* social contexts; the economic, interpersonal and environmental dimensions of their lives. With regard to work capability, symptoms that constitute the diagnosis are simultaneously constitutive of the functional impairment, making a separation along the lines of “condition” and “effects” potentially difficult to articulate. Work-related effects of mental distress are also context dependent, influenced by structural and relational factors within the workplace, and cannot be established absent of a specific work environment. Finally, for people experiencing mental distress, there is invariably a complex multiplicity of socioeconomic difficulty and disadvantage contributing to their work limitations, some of which may have a contributory role in mental distress, and all of which require consideration and support alongside attention to mental health per se. These three levels of separation - which we suggest would benefit from *integration *in the WCA - are depicted in Fig. 2.

The focus of the WCA on *health* impairments does not allow space for claimants to describe the broader range of personal, practical, social, structural or economic barriers to work that they may be facing. To position poor mental health as the *determining* barrier to work masks the range of complex and interrelated factors that underlie a person’s experience of distress *and* worklessness. Whilst the signs and symptoms of mental distress may make it difficult for someone to carry out a work role consistently, the origins of such experiences are not merely biological disorder, but are often manifestations the result of a complex mesh of socially rooted challenges, threats and hardships which need to be tackled at source if a person is to be able to move towards and into fulfilling and sustainable employment.

Welfare assessment systems that are driven primarily by biomedical understandings of work limitation can obfuscate the multiple interrelated factors that pose barriers to employment. In doing so, they also lead both claimants and welfare practitioners to locate problems within the medical frame. As has been observed in studies of healthcare interactions [68, 69, 70], where welfare systems do not accommodate a social perspective on distress, both the claimant/patient and practitioner have to shape their narratives through the language and constructs of mental “illness”. Thomas et al. [70] demonstrate how “poverty-related distress” becomes medicalised as a means of legitimisation within UK health and welfare systems, with GPs’ “…proffering of mental health diagnoses and treatment to enable the patient to legitimise their welfare claims, even in cases when the GP did not see the issue as inherently medical”. In the USA, major reforms to the welfare system in the mid-1990s have led to a progressive narrowing of entitlement and increasingly medicalised eligibility criteria [71, 72]. Hansen et al.’s [71] ethnographic study revealed how receiving and sustaining a psychiatric diagnosis may be the only way that people can find validation and financial stability in the midst of myriad complex social troubles. Research in the Scandinavian welfare context similarly reveals how medicalised eligibility systems lead welfare professionals to frame social problems as medical issues [73, 74].

Health-related eligibility criteria may drive people to project and internalise a "sick role" [75] as the only viable means of legitimising and securing welfare status. Thus, the mentally ill identity may become a welfare-based “survival strategy” [71] which then inhibits effective steps towards employment. A medicalised framing that rests on entification and foregrounding of psychological impairment thus diverts attention away from a focus on capability and possibility, and may therefore become obstructive at a practical level in assisting people to identify and address the range of work-related support needs and to explore types of occupation that may be feasible and fulfilling alongside and in spite of any ongoing and fluctuating experiences of distress.

How, then, might a “gateway” assessment for benefit entitlement understand and evaluate capacity for work in a way that does not rely (solely) on medicalised notions of mental illness, and instead takes a fuller and more person-centred approach to understanding the experience and effects of mental distress, and its place within a person’s broader social, economic and relational milieu? One possibility might be an assessment process that adopted a less rigid questioning format, and invited a more claimant-led narrative about their personal circumstances and perceived barriers to finding and keeping work. The fact that around two-thirds of WCA decisions are overturned at appeal^6^—where the assessment criteria are identical, but discussion is more unstructured—suggests that, with more time and support to explain their circumstances, claimants’ needs and barriers are better understood.

The adoption of a “real world assessment”, in which capacity for work is assessed against specific job roles, wider personal circumstances including education and skills, and (in some cases) the realities of local labour markets [41] would—to some extent—address this paper’s concerns about separation of experience from context. These approaches can be found in international models including the Netherlands, Sweden and Denmark [41, 42] and attend to our second tier of separation, at which the individual’s capacity for work is problematically decontextualised from any given job role. However, to the extent that this paper offers provocation rather than concrete policy proposals, we go further than these regimes’ closer matching of health impairment to labour market options, and suggest that health be more radically decentred from its place as the sole determining criteria in establishing work capacity. The vision we put forward here is one in which the conversation between claimant and assessor at the point of seeking benefit support is not necessarily or pivotally anchored around health impairment, but approaches the individual in a holistic way, taking account of health *and non-health* influences on work capacity, and—in the case of mental health—allowing a framework in which the claimant can convey their experience of distress in their own terms, which may or may not utilise a biomedical framing.

The propositions put forward in this paper lead inescapably to a number of challenging questions about the policy implications that would necessarily follow. Key issues that circle above, and are inextricably connected to, the propositions we put forward are (i) implications for more radical and fundamental reform of conditionality-based welfare regimes, and associated debates about the pros and cons of a Universal Basic Income [76, 77, 78]; and (ii) critical questions about how to assess and support the extra costs and labour market disadvantage experienced by disabled people and those living with long-term health conditions, if this were decoupled from income-replacement benefit groupings. In this regard, it is essential to acknowledge that disabled people’s organisations have critiqued and expressed concerns about proposals for Universal Basic Income [79, 80], and that the erasing of health-related group distinctions would be experienced as highly threatening for many people. As expressed by the Commission on Social Security [81], “Entirely removing a disability category from out-of-work benefits is extremely dangerous because it fits so well with an agenda to dismiss and deny that disabled people experience any material barriers to employment and are unable to support ourselves through paid employment—as such it could be described as ideologically dangerous”.

We have focussed in this paper on the *initial* assessment of eligibility for health-related benefits. However, our arguments clearly have relevance to design and focus of the welfare-to-work interventions that follow. Whilst the “independence” of those conducting the WCA is purported to be a virtue of the system [9], the disconnection of assessment of capacity for work from the support that follows is perhaps therefore a fourth problematic separation. Reforming the WCA in a way that focussed on identifying what a person *would need in order to feel able* to work, and which was more closely integrated with person-centred, holistic employability schemes of the kind that have been demonstrated in a growing number of local initiatives [58, 82, 83, 84, 85, 86], might create a better experience for claimants and leading to more productive employability outcomes.

## Conclusion

Government strategies going back at least three decades (Department of Health [87] cited in Buck [88]) have recognised that mental health is influenced by social circumstances, including family, education, housing and employment. Yet welfare assessment processes continue to treat a “mental health condition” as a decontextualised and entified barrier in its own right, when in many cases these are responsive manifestations of distress caused by a person’s social context and have different implications for work capacity depending on environment. We have argued here that welfare systems founded on a medicalised conceptualisation of “mental health conditions” as discrete drivers of work limitation obfuscate the holistic range of social and structural barriers to work (which may, for some people, be the more instrumental), neglect the critical role of social and interpersonal context in assessing work capacity, and in doing so contribute to less effective and even counterproductive welfare-to-work outcomes.

Extant research illustrates the ways in which medicalised frameworks shape people’s interactions with health and welfare systems. Claimants and welfare operatives are aware of the socioeconomic factors that frequently underlie distress, and are conscious of the misdirected actions that current systems necessitate. However, where health impairment is the passport to welfare support, all parties are bound by current institutional processes to approach work-related barriers through a predominantly medicalised framing. In essence, a person must frame their experience of distress in  medical terms in order to be recognised and supported by the welfare system. The assessment system as currently designed pays little regard to acknowledging or supporting underlying barriers to employment that go beyond the manifest “mental health” symptomology. The benefits system instead has become a battleground on which to prove (or disprove) severity of impairment, rather than an arena in which the totality and complexity of people’s lives can be understood and supported in a diverse range of more effective ways.

This discussion is timely, as the UK Government conducts a Select Committee inquiry into the benefit assessment process and the Department for Work and Pensions (DWP) progresses its Health Transformation Programme, key objectives of which include to “improve the trust and transparency in the assessment process … focus[ing] on improving the claimant experience”.^7^ As noted by the Social Security Advisory Committee [89] “There have been many changes to the benefit system in terms of how it impacts disabled people over recent decades. But none of them have led to a significant improvement in economic activity”. We have suggested here that a more holistic assessment of work capacity—a different kind of conversation, that considered not only the (fluctuating) effects of psychological distress but also the range of personal, social and economic circumstances impacting an individual—would offer a less distressing and ultimately more productive approach to understanding work capability. Such a shift would reduce the need to focus on medically framed incapacity for work and open up space in encounters for a more empowering focus on capacity, capabilities, aspirations, and what types of work are (or might be) possible.

Debates about the concrete policy proposals that might follow from a more holistic and demedicalised approach to work capacity assessment lie beyond the scope of the present paper, but we hope that the ideas we have raised will prompt more applied policy discussions. Here, we have sought to explore whether a less medical-centric framing of the current assessment system may, potentially, lead to more productive, less distressing, and more epistemically just [9, 90] experiences for benefit claimants who are experiencing mental health problems. A more holistic and socially informed assessment, that took into account the claimant’s perspective on the social origins of their distress and a person-centred exploration of its effects, may lead to claimants feeling more heard and their circumstances being better understood by those making assessments of their capacity for work. It would also enable the uncovering of the complex combination of personal, practical, social, structural and economic factors that collectively constrain an individual’s ability to engage in paid employment at certain times of their life. Such an approach would not overcome the fundamental harms brought about by a conditional welfare system, but they may offer a stepping-stone towards a less distressing model of welfare-to-work intervention that edges closer to the original intentions of the welfare state.

## Data Availability

There are no data obtained for this report.

## Footnotes

1 The UK welfare system permits reduced work-related obligations for a limited set of other circumstances, including caring responsibilities for a severely disabled person or for a child under three years. This paper is concerned with the assessment pathway for those seeking working-age benefits on the grounds of personal ill health, specifically mental health.

2 We thank Dr Rochelle Burgess for introducing this notion of “separation” as a way to frame our analysis.

3 Whilst claimants the first two groups receive the same level of monetary benefit, those in the *limited capability for work or work-related activity* group also receive a higher rate of benefit, as well as reduced conditionality. The provision of higher value benefits in respect of the additional costs of ill health and disability is a significant aspect of any discussion around changes to welfare policy, but one which lies beyond the scope of the current article. However, we acknowledge this as a as key area for further debate, which inescapably arises from the propositions we put forward here.

4 The full UC50 form can be viewed here: https://www.gov.uk/government/publications/uc50-form-universal-credit-capability-for-work-questionnaire.

5 Space has not permitted discussion of the role of “demand side” factors, in terms of stigma and employer reticence to recruit, support or retain employees who experience mental health difficulties [59], nor the quality of employment that may be available to individuals moving off benefits and the potentially damaging effects of precarious and insecure work on mental health [60]. However, we recognise the inextricable connection between our present discussion and the key issues of “good work” and mentally healthy workplaces.

6 https://www.gov.uk/government/statistics/esa-outcomes-of-work-capability-assessments-including-mandatory-reconsiderations-and-appeals-june-2022/esa-work-capability-assessments-mandatory-reconsiderations-and-appeals-june-2022#appeals-clearances-and-outcomes-for-initial-esa-claims-which-started-up-to-march-2021.

7 At time of writing, in light of a post-pandemic increase in economic inactivity, there has also been debate within both of the UK’s main political parties around “radical overhaul” of the benefits assessment system and even “scrapping the WCA” entirely: https://www.theguardian.com/society/2023/jan/12/disability-benefits-shake-up-still-claim-return-work.
